# Hypercellular Cystic Mucinous Carcinoma of the Breast With a Growth Pattern of Encapsulated Papillary Carcinoma

**DOI:** 10.7759/cureus.95549

**Published:** 2025-10-27

**Authors:** Takahiro Mase, Takeshi Hasegawa, Chiaki Takagi, Masahito Nawa, Hideki Mori

**Affiliations:** 1 Breast and Endocrine Surgery, Ogaki Tokushukai Hospital, Ogaki, JPN; 2 Surgery, Ogaki Tokushukai Hospital, Ogaki, JPN; 3 Clinical Laboratory, Ogaki Tokushukai Hospital, Ogaki, JPN; 4 Breast Oncology, Nawa Clinic for Breast Oncology, Ogaki, JPN; 5 Pathology, Ogaki Tokushukai Hospital, Ogaki, JPN

**Keywords:** breast, cystic lesion, encapsulated papillary carcinoma, neuroendocrine tumor, surgery

## Abstract

We report the case of an 82-year-old woman who developed a 70-mm cystic lesion in the breast. The cyst contained a papillary mass with numerous delicate fibrovascular stalks within a cystic space surrounded by a thick fibrous capsule. No myoepithelial cells were identified along the fibrovascular stalks. The neoplastic epithelial cells were arranged in micropapillary and cribriform structures. The tumor was composed of cells ranging from spindle-shaped to polygonal, with intermediate-grade nuclei. There were no densely cellular or solid nests of neoplastic cells. In some areas, tumor cells were also present in the cyst wall. Initially, the tumor was considered an encapsulated papillary carcinoma (EPC). However, more than 70% of the neoplastic cells were positive for synaptophysin and neuron-specific enolase (NSE), both markers of neuroendocrine differentiation. Therefore, the tumor was judged to represent a neuroendocrine neoplasm. Additional Alcian blue staining demonstrated a small amount of mucin in the extracellular space and lumens. Accordingly, the present case was diagnosed as a mucinous carcinoma (MC) of hypercellular type with neuroendocrine differentiation. To the best of our knowledge, a cystic MC of the breast exhibiting growth patterns of EPC has not been previously described.

## Introduction

Mucinous carcinoma (MC) of the breast is an invasive breast carcinoma (IBC) characterized by clusters of epithelial tumor cells suspended in pools of extracellular mucin. MC accounts for approximately 2% of all breast carcinomas and most often occurs in older women [1]. MC is divided into type A and type B. As described by Capella et al. [2], type A MCs are relatively hypocellular, with abundant extracellular mucin, whereas type B MCs tend to be hypercellular and consist of large epithelial clumps that often show neuroendocrine differentiation. Pure MC requires a mucinous component of >90%, while mixed MC has 10%-90%. A mucinous component of <10% should still be mentioned. The transcriptomic features of type A MC differ from those of type B, with the latter showing a gene expression pattern similar to that of neuroendocrine carcinomas [2]. MC is usually positive for estrogen receptor (ER) and progesterone receptor (PR) and positive for androgen receptor (AR) in about 80% of cases. HER2 overexpression and/or amplification is rare in MC but is found in over 10% of MCs with a micropapillary pattern. MC accounts for approximately 2% of all breast carcinomas and typically occurs in older women, with a median age of 71 years [1].

Encapsulated papillary carcinoma (EPC) of the breast is characterized by delicate fibrovascular stalks lined by neoplastic epithelial cells, usually within a cystic space surrounded by a fibrous capsule. The neoplastic epithelial cells, with low- to intermediate-grade nuclei, are arranged in micropapillary and cribriform structures. Myoepithelial cells are typically absent along the papillae and at the periphery of the lesion. EPCs express ER and usually PR, lack HER2, and show a low to occasionally moderate Ki-67 proliferation index. The differential diagnosis includes solid papillary carcinoma (SPC), papillary ductal carcinoma in situ (DCIS), invasive papillary carcinoma, and invasive carcinoma of no special type (NST) [3]. The incidence of EPC is difficult to determine. Less than 2% of breast carcinomas are papillary carcinomas, and only a proportion of these are EPCs [4].

Neuroendocrine tumor (NET) of the breast is an invasive tumor characterized by low- to intermediate-grade neuroendocrine morphology, supported by the presence of neurosecretory granules and diffuse, uniform immunoreactivity for neuroendocrine markers. NETs represent <1% of breast carcinomas and approximately 50% of cases designated as carcinomas with neuroendocrine differentiation. NETs of the breast are defined as epithelial tumors with a morphology similar to gastrointestinal and pulmonary NETs, expressing neuroendocrine markers. Most patients are in their sixth or seventh decade of life [5].

We encountered a rare case of carcinoma developing within an unusually large cystic structure with a thick wall. The neoplasm displayed features of papillary or tubular carcinoma with moderate atypia. Infrequently, the tumor showed carcinoid-like features such as ribbons, cords, and rosettes. Furthermore, more than 70% of the neoplastic cells expressed synaptophysin and neuron-specific enolase (NSE). Accordingly, the case was initially considered a NET with an EPC-like growth pattern. However, additional Alcian blue staining demonstrated a small amount of mucin in the extracellular spaces and lumens, suggesting that the tumor is an MC of hypercellular type with neuroendocrine features.

## Case presentation

**Figure 1:**
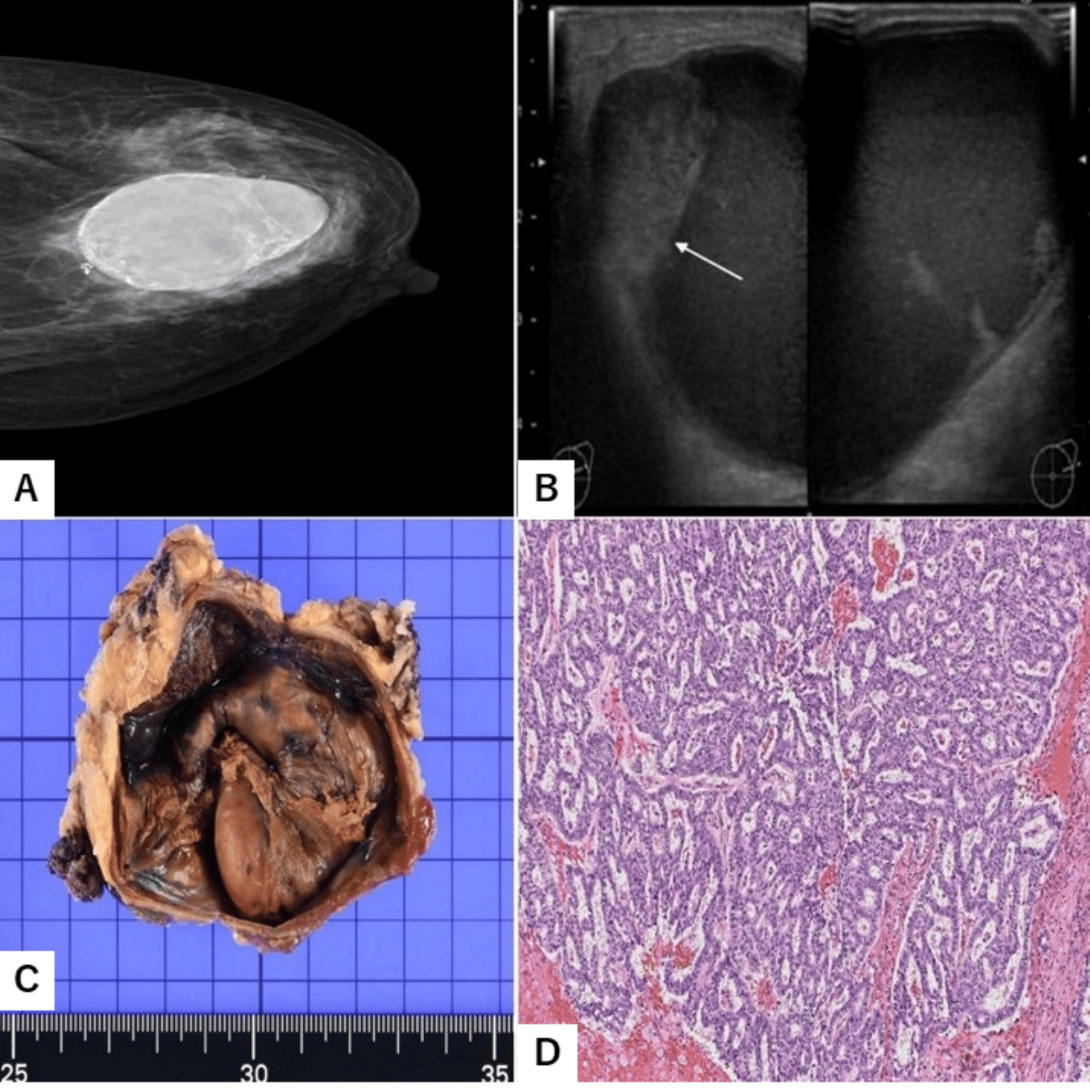
Cystic mucinous neoplasm of the breast (A) Digital mammogram showing a high-density, oval mass in the upper-outer region. (B) Ultrasonography showing a large cystic structure with a nodular mass inside (arrow). (C) Macroscopic appearance of the lesion; adjacent breast adipose tissue is attached to the fibrous capsule. (D) Histology of the neoplasm showing micropapillary and cribriform structures. Some areas were examined with Alcian blue staining. H&E stain, 5 × 10.

An 82-year-old woman noticed a mass in her left breast and soon visited a breast oncology clinic. Her medical history included depression, type II diabetes mellitus, hypertension, femoral fracture, and osteoporosis. She had no previous history of malignant neoplasms. At the clinic, a soft mass, approximately 7.0 cm in size, was confirmed. Mammography revealed a high-density, oval mass in the upper-outer quadrant of the breast (Figure 1A). The patient was referred to the Department of Endocrine Surgery at Ogaki Tokushukai Hospital for further evaluation of the left breast lump. Ultrasonography performed at our hospital demonstrated a 7.0-cm cystic structure (Figure 1B) containing a nodular mass (arrow). Aspiration cytology of the cyst fluid showed possible ductal cells with mildly increased nuclear chromatin and the presence of multiple phagocytes. The cytologic diagnosis was Atypia/C3 (Papanicolaou classification). Lumpectomy of the left breast lesion was performed one month after the initial presentation. The excised mass measured 7.0 × 6.0 × 5.0 cm.

The macroscopic appearance of the lesion removed during surgery is shown in Figure 1C. Multiple nodular masses were located within a cystic space surrounded by a thick fibrous capsule, which was attached to the breast adipose tissue. Histologically, the neoplastic cells were arranged in micropapillary and cribriform patterns, filling the gaps between adjacent papillae (Figure 1D). The morphology suggested papillary carcinoma, with tumor cells showing intermediate-grade nuclei. Occasionally, neoplastic cells were also observed within the fibrous capsule; however, frank invasion was ruled out, as no neoplastic elements clearly extended beyond the capsule. Neoplastic cells were not identified in the adjacent adipose tissue, and there was no evidence of invasion into the surrounding connective tissue. In addition, no DCIS component was identified in the present case.

CK14 staining demonstrated the absence of myoepithelial cells in the tumor. Strong and diffuse ER staining was observed in the neoplastic cells, while PR and HER2 expression were negative. 

**Figure 2:**
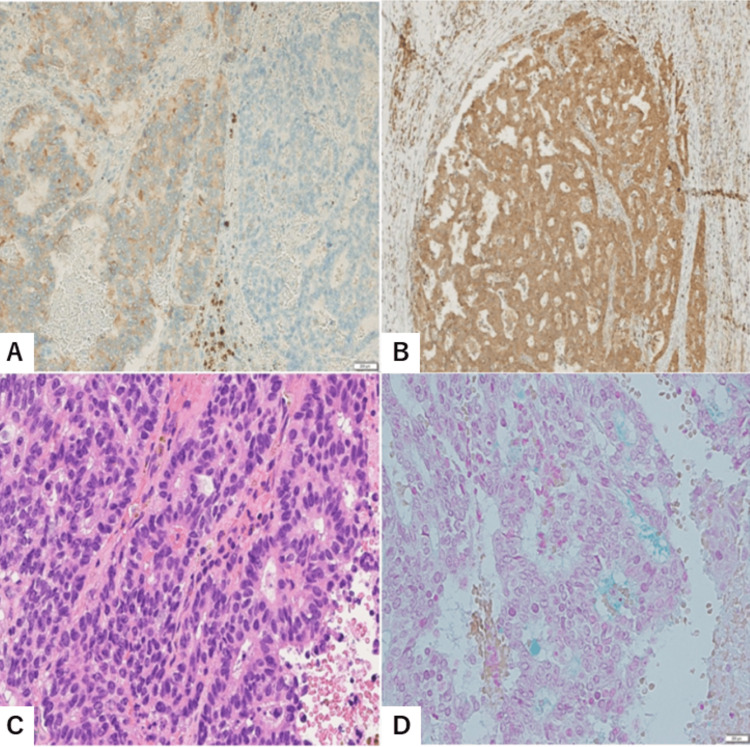
Histology of the mucinous carcinoma (A) Immunostaining for synaptophysin showing a positive response in tumor cells (left) and a negative response in other tumor areas with similar morphology (right). Original magnification: 5 × 20. (B) Immunostaining for neuron-specific enolase (NSE) showing diffuse expression in nearly all tumor cells. Original magnification: 5 × 10. (C) Carcinoid-like pattern of tumor cells exhibiting nuclear pleomorphism, overlapping, and dense chromatin, suggesting a hypercellular subtype of mucinous carcinoma. Original magnification: 5 × 40. (D) A small amount of mucin present in the extracellular space around the tumor nests and lumens. Alcian blue stain, 5 × 40. NSE: neuron-specific enolase.

By immunostaining for synaptophysin, more than 70% of the tumor cells showed positive reactivity (Figure 2A), whereas chromogranin A, another neuroendocrine marker, was negative. In addition, nearly all tumor cells displayed diffuse expression of NSE (Figure 2B). In some areas, the tumor exhibited carcinoid-like features, including ribbons, cords, and rosettes (Figure 2C). The Ki-67 labeling index of the tumor cells was 10%-20%, and the mitotic count was 5-10 per 10 high-power fields. Tumor necrosis was not observed. These findings strongly suggested that the neoplasm was a grade 2 neuroendocrine tumor. To further differentiate it from MC, Alcian blue staining was performed to assess the presence of extracellular mucin. The staining revealed a small amount of mucin in the extracellular spaces around the tumor nests and within the lumens, suggesting that the neoplasm was a mucinous carcinoma of hypercellular type (Figure 2D). Following surgery, the patient has remained healthy with no symptoms or evidence of breast carcinoma recurrence. No malignant neoplasms have been identified in other organs, indicating that the cystic neoplasm of the breast was not metastatic.

## Discussion

The present case was finally diagnosed as MC, hypercellular type (type B). According to recent criteria, such a case is classified as IBC-NST (MC, hypercellular type with a mucinous component < 10%) [6]. As described by Capella et al. [2], type B MC shows a transcriptomic gene expression pattern similar to that of neuroendocrine carcinomas. They also noted that MC of the breast is not a single homogeneous entity but consists of two main variants distinguishable on structural and cytological grounds, as well as a smaller transitional type. One variant is typically argyrophilic and contains dense-core granules similar to those seen in endocrine tumors. Both endocrine and amphicrine cells have been identified in this variant. There is also a recognized relationship between this endocrine variant of MC and other argyrophilic (so-called carcinoid) carcinomas of the breast. 

Lacroix-Triki et al. [7] reported that pure MCs show relatively low genetic instability and less frequently exhibit gains of 1q and 16p or losses of 16q and 22q compared with grade- and ER-matched invasive ductal carcinoma (IDC)-NSTs. In contrast, no pure MC displayed concurrent 1q gain and 16q loss, a hallmark genetic feature of low-grade IDC-NSTs. Barbashina et al. [8] described that, unlike ordinary pure MCs, 20% of mucinous micropapillary carcinomas of the breast were positive for HER2, and 23% were p53 positive. More than half (60%) of mucinous micropapillary carcinomas of the breast demonstrated lymphovascular invasion. Meanwhile, Pusztai et al. [9] reported that the mucinous phenotype was associated with the expression of both immunostimulatory and inhibitory genes, consistent with lymphocytic infiltration and the expression of enzymes involved in mucin production.

The present case demonstrated a unique cystic structure. Invasive cystic hypersecretory carcinoma is a rare subtype of breast cancer that also exhibits cystic structures [10]. However, that lesion typically consists of multiple variable-sized cysts and ducts filled with thyroid colloid-like eosinophilic secretions. The entity most closely related to the present case may be mucinous cystadenocarcinoma, which is composed predominantly of tall columnar cells with abundant intracytoplasmic mucin and closely resembles mucinous cystadenocarcinomas of the ovary and pancreas [11]. Nonetheless, the present case is not multicystic, and tall columnar cells were not observed. Accordingly, identical cases have not been reported in the literature. Cystic neuroendocrine tumors have, however, been recognized in the pancreas and other organs such as the liver and tailgut [12-14]. Therefore, examination for possible coexistence of MC within such neuroendocrine tumors may be warranted.

The pathogenesis of the large single cyst in the breast remains unclear. The authors propose two possible mechanisms. One possibility is that cells with the potential for differentiation toward neuroendocrine and mucinous neoplasms migrated into a congenital cyst or a pre-existing benign lesion such as a cystic papilloma. The second possibility is that cyst formation resulted from tumor growth with secondary cystic degeneration due to repeated hemorrhage or necrosis/apoptosis. Based on the findings, the former process seems more likely, as the cyst in the present case was quite large and had a thick capsule. Furthermore, there was no evidence of massive hemorrhage or necrosis within the cystic neoplasm.

## Conclusions

An 82-year-old woman developed a neoplasm composed of cells arranged in micropapillary and cribriform structures located within a large cyst in the breast. Based on the macroscopic appearance, histology, and growth pattern, the neoplasm was initially considered an EPC. However, a small amount of mucin was identified in the extracellular spaces and lumens, and more than 70% of the neoplastic cells expressed synaptophysin and NSE. Accordingly, the cystic neoplasm was diagnosed as a hypercellular MC with neuroendocrine differentiation. To our knowledge, such a case of cystic MC of the breast has not been previously described.

## References

[1] Wen HY, Desmedt C, Reis-Fiho JS, Schmitt F (2018). Mucinous carcinoma. WHO Classification of Tumours, Breast Tumours, 5th Edition.

[2] Capella C, Eusebi V, Mann B, Azzopardi JG (1980). Endocrine differentiation in mucoid carcinoma of the breast. Histopatol.

[3] Mac Grogan G, Collins LC, Lerwill M, Rakha EA, Tan BY (2018). Encapsulated papillary carcinoma. WHO Classification of Tumours, Breast Tumours, 5th Edition.

[4] Lefkowiz M, Lefkowiz W, Wargotz ES (1994). Intraductal (intracystic) papillary carcinoma of the breast and its variants: a clinicopathological study of 77 cases. Human Pathol.

[5] Rakha EA, Reis-Fiho JS, Wu Y (2018). Neuroendocrine tumour. WHO Classification of Tumours, Breast Tumours, 5th Edition.

[6] Rakha EA, Allison KH, Bu H (2018). Invasive breast carcinoma of no special type. WHO Classification of Tumours, Breast Tumours, 5th Edition.

[7] Lacroix-Triki M, Suarez PH, MacKay A (2010). Mucinous carcinoma of the breast is genomically distinct from invasive ductal carcinomas of no special type. J Pathol.

[8] Barbashina V, Corben AD, Akram M, Vallejo C, Tan LK (2013). Mucinous micropapillary carcinoma of the breast: an aggressive counterpart to conventional pure mucinous tumors. Human Pathol.

[9] Pusztai L, Sotiriou C, Buschholz TA (2003). Molecular profiles of invasive mucinous and ductal carcinomas of the breast: a molecular case study. Cancer Genet Cytogenet.

[10] Sun J, Wang X, Wang C (2019). Invasive cystic hypersecretory carcinoma of the breast: a rare variant of breast cancer: a case report and review of the literature. BMC Cancer.

[11] Koenig C, Tavassoli FA (1998). Mucinous cystadenocarcinoma of the breast. Am J Surg Pathol.

[12] Hurtado-Pardo L, Cienfuegos JA, Ruiz-Canela M, Panadero P, Benito A, Herandez-Lizoain JL (2017). Cystic pancreatic neuroendocrine tumors (cPNETs): a systemic review and meta-analysis of case series. Rev Esp Enfarm Dig.

[13] Shetty PK, Baliga SV, Balaiah K, Guana PS (2010). Primary hepatic neuroendocrine tumor: an unusual cystic presentation. Ind Pathol Microbiol.

[14] Guo W, Deng M, Chen Q (2024). A neuroendocrine tumor arising in a tailgut cyst: case report and literature review. Int J Surg Case Rep.

